# *Announcement*: Global Handwashing Day — October 15, 2017

**DOI:** 10.15585/mmwr.mm6640a6

**Published:** 2017-10-13

October 15, 2017, marks the 10th annual Global Handwashing Day. This observance helps to increase awareness and understanding of handwashing with soap as an effective and affordable way to prevent disease around the world.

Handwashing with soap has an important role in child survival and health. Approximately 1.4 million children aged <5 years die each year from diarrheal diseases and pneumonia, the top two causes of death among young children globally (*1*). Handwashing with soap can reduce the incidence of diarrhea and respiratory infections among children in this age group by approximately 30% and 20%, respectively (*2*,*3*).

Although persons around the world clean their hands with water, few use soap to wash their hands, because soap and water might be less accessible in developing countries. Even when soap is available, it might be reserved primarily for laundry and bathing instead of for handwashing. Washing hands with soap removes germs more effectively than water alone (*4*), and can help in preventing diseases and saving lives. Additional information on Global Handwashing Day and handwashing in general is available from CDC at https://www.cdc.gov/handwashing. Information on water-related hygiene is available at https://www.cdc.gov/healthywater/hygiene.
